# The Puerperal Diseases

**Published:** 1874-02

**Authors:** 


THE PUERPERAL DISEASES: Clinical Lectures delivered at Bellevue Hospital. By Fordyce Barker, M.D., Clinical Professor of Midwifery and the Diseases of Women in the Bellevue Hospital Medical College, etc., etc. New York: D. Appleton & Co., 549 and 551, Broadway, N. Y., 1874.

The author has for twenty years been giving clinical lectures on "Midwifery, the Puerperal and other Diseases of Women," and the volume before us "is made up substantially from phonographic lectures" delivered during this time. The volume opens with a chapter devoted to puerperal convalescence. Puerperal convalescence, defined by Prof. Murphy, is—"1. The interval between the birth of the child and the commencing secretion of milk; 2. The period during which the function of lactation rises to its highest point of activity; 3. The period occupied in restoring the uterus to its original condition previous to conception." The author enters into details in his teachings upon puerperal convalescence giving specific directions for its management and treatment. He notices the frequent retention of urine—after-pains—the lochia and secondary hemorrhage, with the various producing causes of the latter, so important to be well understood in order to afford the patient the necessary relief. He recommends when the "second stage of labor is too rapid or too prolonged," the use of a full dose of ergot "just as the delivery of the child is taking place." This practice we have followed in many instances—especially when apprehensive of post-partum hemorrhage. We have found, however, that it increases the after-pains in those who usually suffer most from them. For these after-pains Dr. Barker's "favorite prescription" is "ten grains of Tully's powders,* repeated, if necessary, in four or five hours." We have generally found Meigs' camphor-julep to answer in the majority of cases, with tr. opium and tr. camphor used warm on folds of flannel and applied over the uterus. In very severe cases he employs "quinine (5 to 10 grains) internally, and the application of chloroform liniment,† externally," which, he says, is so successful that it is "rarely needed for more than a day or two."

For offensive lochial discharges he recommends carbolic acid‡ injection, and remarks, that in private practice he is "in the habit of directing the injection somewhat weaker, for the first few days in all cases." The general directions, in every particular, are just such as the practitioner would likely seek for, and so amply given as to cover the periods of puerperal convalescence. Under the head of "Diet of Puerperal Women," after giving many practical suggestions, he advises the practice of giving "puerperal women as good, nutritious food as she has an appetite for and can easily digest and assimilate." He speaks of the common practice of giving laxatives to women after confinement. He does not advise the employment of "castor oil," and asserts he has seen "severe suffering from piles" as a consequence of its use. He prefers, when torpor of the bowels alone exist, the warm water and castile soap enema, but remarks, that he "waits for some indication of the necessity of a laxative before prescribing one." He makes some practical remarks on hemorrhoids in pregnant and puerperal women, which we cannot notice here, more than to call attention to his use of aloes for these cases. In the chapter on puerperal convulsions he devotes considerable space to the discussion of the cause and pathology of the disease. He does not accept the proposition of Dr. Braun, of Vienna, "of uræmic intoxication arising from Bright's disease," and shows that in many cases there were no

*Tully's Powders.—R. Pul. g. camph.; cretæ, pp.; pulv. glycyrrh, aa gr. 20; morphiæ sulph., gr. 1. M. Dose same as Dovers powder.

†R Chloroform, 1 ounce; lin. sapo. co, 6 ounces. M.

‡R Acidi carbolici glacial, 1 ounce; glycerine, 1 ounce; aquæ puræ, 8 ounces. M S. A tablespoonful in eight ounces of warm water, twice a day, as a vaginal injection.

lesions of the kidneys—that in "marked cases of albuminuria during pregnancy, convulsions do not occur;" and convulsions have occurred when no albumen could be discovered in the urine. He thinks the true pathology of the disease may be explained by the "direct connection between the nerves of the uterus and the renal ganglia"—a discovery made by Frankenhaueser, of jena. The prophylactic treatment is given, which we must omit, simply referring to his recommendation of the use of chloroform during the labor and an early delivery—not objecting to the loss of "a moderate amount of blood." For the general treatment he recommends 20 grains of jalap and 10 grains of calomel, between the attacks, as a purge—giving a grain of elaterium in a third of a teaspoonful of butter, if she be comatose. To arrest or prevent the convulsions, he administers chloroform by inhalation; and having, by these means, "overcome immediate danger," he administers "a full dose of morphia" hypodermically. He has been disappointed in chloral in these cases. He advises the dilation of the cervix to hasten the labor, when it does not spontaneously do so, and in the cases which are not benefitted by the treatment above given.

In the chapter on lacation, he gives his treatment of sore nipples, which so often is the cause of mammary abscess—a synopsis of which is given in another part of the RECORD. The chapter on Mastitis and Mammary Abscess is one of much interest. It very often becomes a question of importance to the practitioner how to best meet an "accident" so common and painful. We cannot do our author justice by any condensation of his treatment—the entire chapter must be read for this purpose. For puerperal mania, aside from other indications for treatment, our author relies mainly upon chloral, and seems not to have used, or overlooked, cimicifuga, so highly spoken of by Sir Jas. Y. Simpson. Of the other chapters—on Relaxation of the Pelvic Symphyses, Phlegmasia Dolens, Puerperal Thombosis and Embolism, Puerperal Phlebitis and Metritis, Peritonitis, Pelvic Cellulitis, Puerperal Septicæmia and Pyæmia—we would like to speak, but our limits deny us the privilege. The volume closes with two most admirable and extended chapters on puerperal fever; in the first of which he makes his "confession of faith," and in the other gives mainly the principles, of his treatment. He relies upon veratrum viride in reducing the fever as the great remedy in the disease, with other adjuvants as may be required.

Having devoted so much space to this valuable work, we close by saying we can and do heartily commend it to our readers. It is a book they will appreciate and value. G.

